# Medial entorhinal cortex plays a specialized role in learning of flexible, context-dependent interval timing behavior

**DOI:** 10.21203/rs.3.rs-2681599/v1

**Published:** 2023-04-17

**Authors:** Erin R. Bigus, Hyun-Woo Lee, Jiani Shi, James G. Heys

**Affiliations:** 1 Interdepartmental PhD Program in Neuroscience, University of Utah; 2 Department of Neurobiology, University of Utah, Salt Lake City, UT 84112, USA

## Abstract

In order to survive and adapt in a dynamic environment, animals must perceive and remember the temporal structure of events and actions across a wide range of timescales, including so-called interval timing on the scale of seconds to minutes^1,2^. Episodic memory (i.e. the ability to remember specific, personal events that occur in spatial and temporal context) requires accurate temporal processing and is known to require neural circuits in the medial temporal lobe (MTL), including medial entorhinal cortex (MEC)^3-5^. Recently, it has been discovered that neurons in MEC termed time cells, fire regularly at brief moments as animals engage in interval timing behavior, and as a population, display sequential neural activity that tiles the entire timed epoch^6^. It has been hypothesized that MEC time cell activity could provide temporal information necessary for episodic memories, yet it remains unknown whether the neural dynamics of MEC time cells display a critical feature necessary for encoding experience. That is, whether MEC time cells display context-dependent activity. To address this question, we developed a novel behavioral paradigm that requires learning complex temporal contingencies. Applying this novel interval timing task in mice, in concert with methods for manipulating neural activity and methods for large-scale cellular resolution neurophysiological recording, we have uncovered a specific role for MEC in flexible, context-dependent learning of interval timing behavior. Further, we find evidence for a common circuit mechanism that could drive both sequential activity of time cells and spatially selective neurons in MEC.

The nervous system must perceive and learn the duration of events and actions to successfully guide adaptive behavior. This is true across a range of timescales, from millisecond timing which allows precise execution of timed movements, to circadian timing which mediates sleep-wake cycles^1,2^. During waking hours, animals heavily rely on the intermediate scale of interval timing (timing on the order of seconds to minutes) to plan and execute actions across a wide range of behavior, including foraging, mating, prey capture and avoidance^7-9^. Prior work to understand the neurobiological basis of interval timing has largely focused on relatively simple timing tasks and has established that circuits in the basal ganglia, frontal, and parietal cortices are necessary for learning and producing such interval timing behavior^10-17^. Many of the identified circuits are key nodes of the procedural memory system. In contrast, medial temporal lobe (MTL) structures, including the hippocampus and entorhinal cortex, are necessary for encoding episodic memory which also requires accurate temporal coding^3-5^. Recently, there has been accumulating evidence to suggest the medial entorhinal cortex (MEC) is well-suited to encode interval time in the MTL memory system. MEC contains time cells that fire regularly at discrete moments as rodents learn and report temporal durations on the scale of seconds^6,18^. As a population, different MEC time cells fire regularly at different moments, like the second hands of a clock, thereby forming a sequence of neural activity, tiling the entire interval timing epoch.

During memory guided navigation, so-called grid cells in MEC and place cells in the hippocampus fire when animals visit particular locations with an environment^19,20^. Over 50 years of research has demonstrated that the coactivity of populations of these spatially selective neurons likely plays a key role in episodic memory by forming a unique “cognitive map” for distinct environments^21,22^. Perhaps the most critical observation in support of this idea demonstrates that place cells and grid cells reorganize their firing fields as animals navigate across different environments, thereby forming a unique map for different spatial contexts^23,24^. By analogy to such spatial cells, MEC time cells might play a key role in episodic memory by providing a unique representation of time capable of encoding distinct temporal experiences. However, it remains unknown whether MEC time cells display this critical feature of context-dependent time coding. To address this question, we developed a novel temporal delayed nonmatch to sample (tDNMS) task that requires context-dependent timing behavior. By performing 2-photon calcium imaging as mice performed the tDNMS task, we uncovered populations of MEC time cells that fire selectively at specific moments in the timing task, and as a population, the sequence spans the entire timing epoch. Remarkably, we find that over the course of learning, these sequences become context-dependent, whereby specific populations of MEC time cells become active on particular trial types. Further, multiple lines of evidence suggest that the activity of MEC time cells plays a causal role specifically in flexible, context-dependent interval timing behavior. Finally, we find evidence for a common circuit mechanism that may support both MEC spatial and time coding. Together these results establish a neural dynamical and circuit mechanism for MEC time cells that is likely to mediate learning of context-dependent interval timing behavior.

## Results

### Mice learn novel tDNMS task using flexible timing behavior

We designed a timing task with the goal of maximally engaging the MTL memory system. Due to the known dependence of delayed nonmatch to sample (DNMS) tasks on MTL regions^25-27^, we adapted this structure to create a temporal delayed nonmatch to sample (tDNMS) task. In each trial of the tDNMS task, mice report whether the duration of pairs of successively presented stimuli differ. Because mice heavily rely on olfaction, we built a flow dilution olfactometer^28^ and signaled stimuli via a single odorant (isoamyl acetate). We validated our system by ensuring that the concentration of the odorant remains constant over the course of 45 mins during a full training session and that the odor concentration can be rapidly controlled and perceived by the mice as reported through sniffing behavior (Figure S1b,c).

**Figure 1.**
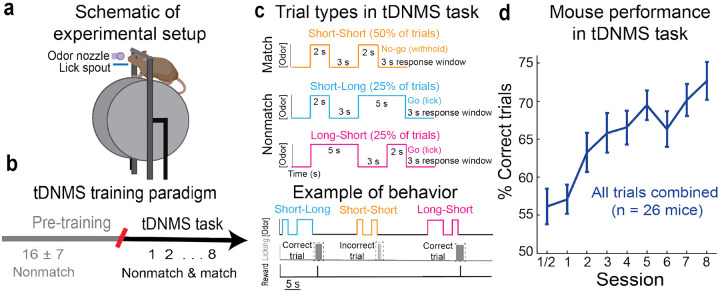
Mice learn a novel tDNMS task. **a.** Simplified experimental set-up. **b.** Overview of training paradigm. Mice are first pre-trained to lick at the offset of nonmatched trials to trigger reward delivery. Upon reaching criteria after an average of 16 sessions, mice begin 8 training sessions in the tDNMS task, where matched trials are introduced. Number of sessions shown as mean +/− standard deviation. **c.** Trial types and example behavior. The tDNMS task consists of 3 types of trials defined by stimulus durations: short-short, short-long and long-short. To perform the task correctly, mice must lick in the response window following non-matched trials and withhold licking in matched trials. **d.** Percent correct for all trial types for each session, averaged across all mice (mean +/− sem; n = 26 mice).

In each trial of the task, a water-restricted, head-fixed mouse (Figure 1a) is presented with two successive stimuli for either a short (2s) or long (5s) duration, separated by a brief 3s interstimulus interval (ISI). The tDNMS task consists of three trial types defined by each stimulus duration: short-long, long-short, and short-short trials. Using a “Go/No-Go” strategy, mice are trained to lick to report a nonmatch of durations (“Go trials”; short-long & long-short) and withhold from licking in response to matched durations (No-Go trials; short-short) (Figure 1c). Before beginning the tDNMS task, mice undergo a shaping procedure to learn the trial structure where they are only presented with nonmatched trials (Figure 1b and Figure S1d; see Methods). As mice reach criteria (see Methods), mice begin the tDNMS task, where matched trials are first introduced and equally balanced across match and nonmatch trial types over each session (90 trials/session over ~45 mins). To test whether mice can learn the tDNMS task, we monitored behavioral performance in 26 mice over 8 training sessions (Figure 1d). Mice began at chance performance on session 1, but steadily improved with training, averaging 73.16 ± 2.47% (mean ± s.e.m. for all data unless otherwise reported) correct responses on session 8 (27.16 ± 4.13% change from Session 1 to Sessions 7&8; Repeated Measures ANOVA F_7,25_ = 8.43, p <0.001).

Limiting the task to 3 trial types allowed mice to robustly learn the tDNMS task over 7-8 sessions. However, this design could potentially allow mice to learn the task through a rigid behavioral strategy. For instance, instead of comparing stimulus durations, as intended, mice could learn to disambiguate total trial duration since match and nonmatch trials are different total durations. We therefore ran a series of control experiments to affirm that mice perform the task by attending to each odor duration. (1) By running the task without odor, we first confirmed that mice require olfactory stimuli to engage in the task (2.25 ± 1.01% correct on nonmatched trials without odor) (Figure S1e). (2) To determine if mice use a rigid strategy involving total trial duration, we manipulated the ISI in a subset of probe trials to make nonmatched and matched trials the same duration. This change did not affect performance, showing that mice use each stimulus duration and not overall trail length to perform the task (74.14 ± 6.71% correct responses on standard nonmatch trials, 80.71 ± 6.02% correct responses on nonmatch probe trials; p=0.19 in paired t-test) (Figure S1f). Together, these experiments demonstrate that mice perceive and use each stimulus duration to guide behavior. Mice use the same durations to differentially guide behavior based upon trial type, making the tDNMS task a flexible, context-dependent timing task that mice learn robustly over 7-8 training sessions.

### MEC time cells fire in context-dependent trajectories during tDNMS task

**Figure 2.**
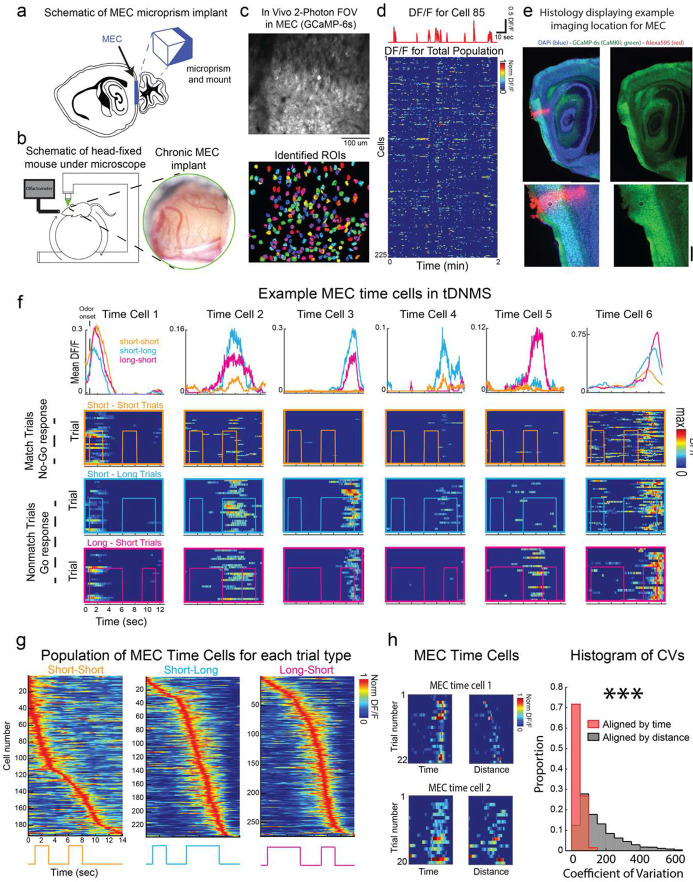
MEC time cells exhibit context-dependent sequential neural activity. **a.** Optical approach for imaging in MEC. **b.** Left, mouse positioned on the cylindrical treadmill and underneath the two-photon microscope. Olfactometer delivers odor near mouse snout. Right, image of MEC prep following chronic implant. **c.** Average image of example FOV labelled with GCaMP-6S (top) and ROIs from this FOV (below). **d.** Example dF/F time series for individual neuron (top) and all ROIs from panel c. **e.** Representative histology. Red pin mark indicates relative location of imaging field. **f.** Example individual MEC time cells. MEC time cells 1, 2 and 6 are active across all trial types (i.e. context independent). MEC time cells 3-5 are active on specific trial types (i.e. context-dependent ). **g.** All MEC time cells sorted for each trial type. **h.** Left, example time cells plotted as a function of elapsed time and distance travelled from trial onset. Right, distribution of coefficient of variation for all MEC time cells, measured as a function of elapsed time and distance travelled from trial onset.

To test the role of MEC in the tDNMS task we applied methods we have previously developed for large-scale cellular resolution two-photon calcium imaging in MEC^64^ (Figure 2a,b). We recorded from populations of layer II MEC neurons expressing GCaMP6s (see Methods; Figure 2c-e), across 6 mice (Field-of-view 430 ± 54 *μm* medial to lateral by 380 ± 45 *μm* dorsal to ventral; depth below the surface 105 ± 8 *μm*) as well-trained mice performed the tDNMS task (16 ± 8 days of training; 74 ± 12% correct trial performance). Across the total population (1194 active neurons), we found that 41 ± 20% of cells exhibited regular time-locked activity at a particular moment in each trial (Figure 2f,g). Consistent with previous reports during interval timing behavior ^6^, we found that different MEC “time cells” were selectively active at different delay times from the start of each trial, forming a regular temporal sequence that spanned the entire trial epoch (Figure 2f,g). During the tDNMS task, mice were free to run on a cylindrical treadmill. Since prior work has shown that many MEC neurons can encode distance travelled ^29,30^, we wondered whether the time-locked activity in MEC time cells might be better explained by distance travelled from trial on-set. In support of the idea that MEC time cells encode elapsed time in the task, and not distance travelled, we found that the vast majority of time cells displayed a smaller coefficient of variation when measuring as a function of elapsed time versus distance travelled from trial onset with CV of elapsed time: 56.97 ± 2.18, elapsed distance: 173.20 ± 7.19, (p = 7.38 e-64, t(381) = −20.59, paired t-test) (Figure 2h).

To perform the tDNMS task, subjects must perceive and compare relative stimuli durations in order to determine trial type (i.e. nonmatch or match) and learn the appropriate response (i.e. Go or No-Go, respectively). We refer to the different combinations of stimuli and trial-type specific responses as “context”. The robust learning and trial structure allowed us to determine whether MEC time cells might encode context in the tDNMS task. First, in well-trained mice, we asked whether MEC time cells display selective responses for specific trial types. By sorting the responses on specific trial types, we found that many MEC time cells were selectively active for only match or nonmatch trials (total context-dependent time cells = 80%; n = 387 out of 484 total time cells), of which 25% (n = 95) were selective for match and 75% (n = 292) were selective for nonmatch) (for examples see Figure 2f: MEC time cells 3-5). Further, among the MEC time cells that were selective for nonmatch trials, we observed several distinct response profiles, with some neurons active on both trial types and some only active on the short-long or the long-short trial type.

### Context-specific sequences support learning of flexible interval timing behavior

Initially, on day 1, mice are not able to accurately disambiguate context, but over several training sessions they learn to respond correctly on 70-80% of trials per session. If context-dependent MEC time cell activity supports task learning, our data should support several predictions. First, we would expect that context-dependent responses should be relatively absent on day 1 and should emerge over the course of learning across sessions. Second, if context-dependent MEC time cell activity supports learning of the correct response, then the coherence of individual time cell activity and/or the regular sequential neural activation across the population should be disrupted on “error trials”, when mice incorrectly report match or nonmatch.

**Figure 3.**
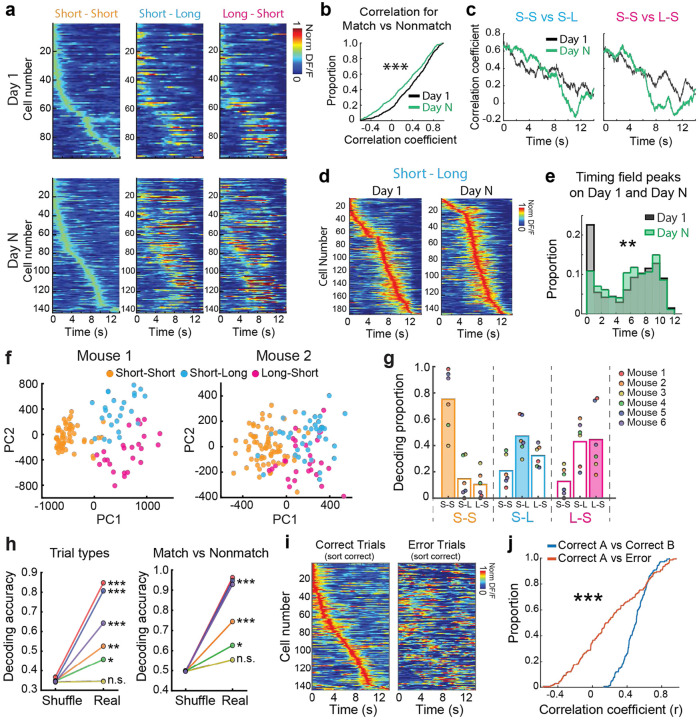
Context-dependent MEC time cell population dynamics support learning of flexible interval timing behavior. **a.** Top, population of MEC time cells (significantly tuned for short-short trials; sorted by short-short trials) for short-short trials (left), short-long trials (middle) and long-short trials (right) on day 1. Bottom, same as above except for day N sessions. **b.** Cumulative distribution for Pearson’s correlation coefficient computed for each MEC time cell’s activity across trial types for day 1 (black) and day N (green). **c.** Left, Pearson’s correlation, plotted as a function of time during trial, for time cell activity for short-short trials versus short-long on day 1 (black) and day N (green). Right, same as left, expect for short-short versus long-short time cell activity. **d.** Sorted sequence of MEC time cells in short-long trials on day 1 (left) and day N (right). **e.** Histogram for peaks of time fields for all MEC time cells on day 1 (black) and day N (green), for all 3 trial types. **f.** PCA plots for individual trials in example sessions. **g.** Trials in each trial type were decoded using LDA, and the decoding result is shown as proportion. Filled bars indicate correct decoding. **h.** Decoding accuracy of each session for trial types (left) and match vs nonmatch (right). Color scheme is same as g. **i.** MEC time cells on short-short correct trials (left) and on short-short error trials on day N of training in tDNMS task. **j.** Cumulative distribution of Pearson’s correlation coefficient computed for MEC time cells on correct (blue) versus error trials (red) (see Methods).

To address our first prediction, we averaged the activity of MEC time cells for each trial type and compared the correlations for each cell’s response across trial types, before and after learning (see Methods). The results demonstrate that the correlations are significantly lower on day N compared to day 1 (day 1: 0.50 ± 0.03, day N: 0.32 ± 0.03, p = 7.83e-05, z = 3.9495, Wilcoxon rank sum test) (Figure 3a,b). These results demonstrate that context-dependent MEC time cell activity is more limited on day 1 and develops to become more distinct over the course of learning. In the tDNMS task, the evidence necessary to disambiguate context accumulates over the course of each trial, with several specific moments during which an “ideal observer” has sufficient information to disambiguate one trial type from another, and ultimately identify the correct trial context. We wondered whether the population dynamics of context-dependent MEC time cells might be informative about this time-dependent decision process, and provide further support that these context-dependent dynamics support learning in the tDNMS task. By sorting MEC time cells according to the time of peak activity on short-short trials, and comparing the correlation of sequences on short-short versus short-long trials, we find that the correlation significantly decreases as a function of time during the trial (significant negative correlations were observed in all plots; SS vs SL Day 1: r = −0.87, SS vs SL Day N: r = −0.95, SS vs LS Day 1: r = −0.92, SS vs LS Day N: r = −0.94, all p-values < 0.0001) (Figure 3a,c). Further, we find that over learning on Day N, the population coherence decreased in larger amount than Day 1 (comparison of correlation between Day 1 and Day N; SS vs SL: Z = 6.05, p < 0.0001, SS vs LS: Z = 2.08, p = 0.0375; Fisher r-to-z transformation). The results demonstrate that over the course of learning, the population of MEC time cells shifts on each trial type to encode later times in the trial, which correspond to moments when there is sufficient information to disambiguate the trial type (peak times on Day 1: 5.55 ± 0.17, Day N: 6.06 ± 0.16, p = 0.0013, two-sample Kolmogorov-Smirnov test; early peak proportion on Day 1: 35 % (n = 168/477), Day N: 26 % (n = 116/442), p = 0.0033, X^2^_(1)_ = 8.7, chi-squared test) (Figure 3d,e). Finally, once the animal learned the task on day N, we tested whether the ensemble activity of time cells on individual trials has sufficient information to accurately decode the temporal context. Trials are well separated according to the trial type in a PCA plot (Figure 3f), and a LDA classifier successfully decoded trial type based on the neural activity of time cells (Figure 3g). We find that the decoding accuracy is much higher than chance level both for trial type (p = 0, 0.002, 0.47, 0.047, 0, 0 for mouse 1~6, respectively) and match vs. nonmatch (p = 0, 0, 0.18, 0.042, 0, 0 for mouse 1~6, respectively)(Figure 3h). This indicates that trial context encoded by time cells is stable and robust in single trials. Interestingly, in some animals all three trial types are clearly separated through distinct neural dynamics (Figure 3f, Mouse 1) while in others the match versus mismatch trials show clear separation but within mismatch, the short-long and long-short trials display more similar dynamics (Figure 3f, Mouse 2). Together, these results all support prediction 1 showing that context-dependent MEC time cell activity emerges over learning, eventually displaying an over-representation at later moments in the task, with large deviations in context-dependent neural trajectories at near key moments in the task when it is possible to distinguish trial type.

To test our second prediction, we compared the average response for each MEC time cell for correct and error trials. At the single cell level, we observed that MEC time cells displayed more consistent activity on correct trials versus error trials, and this was true across all three trial types (Figure S2a). At the population level, we tested whether the specific sequence of MEC time cells for each trial type was less coherent on error trials (see Methods). The results show significantly higher coherence for time cells across randomly selected blocks of correct trials as compared to error trials on day N ([correlation between Correct Trials - Group A and Correct Trials - Group B: 0.56 ± 0.03 vs Correct Trials - Group A and Error Trials: 0.30 ± 0.05, p = 5.998e-05, z = 4.0129, Wilcoxon signed-rank test]) (Figure 3i,j). Thus, the results testing both predictions on the emergence of context-dependent time cell activity over learning and the results on error trials provide support for the hypothesis that context-specific MEC time cell sequences support learning of flexible interval timing behavior.

### Does MEC time cell activity encode absolute time and/or relative time of the tDNMS task?

To answer this question, we recorded MEC time cells in the normal tDNMS task. Then half way through the recording session a longer ISI of 5 seconds was introduced (Figure S2c). Mice responded to the change in the ISI by delaying approach behavior and predictive licking (Figure S2d). We quantified this through the center of mass of velocity and licking (in units of seconds) on normal and probe trials: velocity in S-L trial type: normal (4.73 ± 0.11 s) vs probe (5.67 ± 0.10 s), p < 0.0001, z = 5.41; velocity in L-S trial type: normal (4.92 ± 0.19 s) vs probe (5.20 ± 0.10 s), p = 0.0065, z = 2.72; licking in S-L trial type: normal (13.08 ± 0.07 s) vs probe (14.51 ± 0.06 s), p < 0.0001, z = 8.72; licking in L-S trial type: normal (13.12 ± 0.08 s) vs probe (14.47 ± 0.03 s), p < 0.0001, z = 8.07; Wilcoxon rank sum test. These date indicate that mice perceived the difference in the trial structure during the probe trials. On S-S trials mice did not exhibit clear approach behavior or licking, making it difficult to measure the effects of the probe trials. We found that, on average, the population of MEC time cells activity was delayed in response to the longer ISI (peak times on normal and probe trials: S-S trial types: normal (6.48 ± 0.42 s) vs probe (8.61 ± 0.50 s), p < 0.0001 z = 5.01; S-L trial types: normal (7.13 ± 0.32 s) vs probe (8.71 ± 0.45 s), p < 0.0001, z = 4.36; L-S trial types: normal (8.25 ± 0.42 s) vs probe (9.27 ± 0.43 s), p = 0.0013, z = 3.22; Wilcoxon signed-rank) (Figure S2e,f). These results suggest that rather than absolute time from trial onset, the majority of MEC time cells encode relative time (or “phase”) of the tDNMS task.

### MEC is required to learn flexible, context-dependent timing behavior

**Figure 4.**
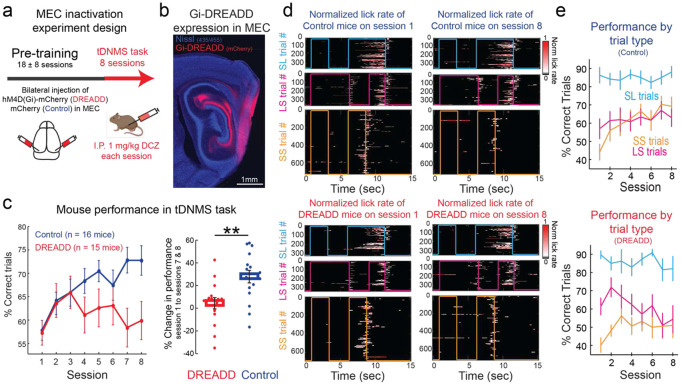
MEC is necessary to learn the tDNMS task. **a.** Experimental paradigm. Mice received bilateral injections of either an inhibitory DREADD (n=15) or control virus (n=16). Mice began pre-training with only nonmatch trials and learned to lick at the offset of second odor stimuli. Following pre-training, mice began the tDNMS task. DREADD agonist DCZ (1mg/kg) was administered via I.P. 5 minutes prior to each of the 8 tDNMS sessions. Pretraining sessions reported as mean +/− s.d. **b.** Sagittal section depicting hM4D(Gi)-mCherry expression in MEC with blue NeuroTrace staining. **c.** Left, performance on the tDNMS task differs for Control (blue) and DREADD (red) mice (Repeated measures ANOVA Group Type x Time F(7,7) = 2.69, p<0.05). Right, percent change in correct response from day 1 to average of days 7 and 8 (p<0.01, unpaired Student’s t-test). **d.** Top, licking behavior during tDNMS task for Control mice on session 1 (left) and session 8 (right) for all 3 trial types. Bottom, licking behavior during tDNMS task for DREADD mice on session 1 (left) and session 8 (right) for all 3 trial types. Consummatory licking following reward delivery is not shown. **e.** Average correct response for Control (top) and DREADD mice (bottom) for all three trial types. Control mice show an improvement in match trials (Repeated measures ANOVA Group Type x Time F(7,15) = 5.93, p<0.001) but DREADD mice do not (Repeated measures ANOVA Group Type x Time F(7,14) = 5.93, p<0.15). Bars indicate mean +/− SEM.

The emergence of context-dependent MEC time cells provides a potential neural dynamical mechanism that could underlie tDNMS learning. To causally test if MEC activity is necessary to learn the tDNMS task, we used a chemogenetic approach to inhibit MEC during task learning (Figure 4a; Figure S3). We first validated our approach by local injection of AAV expressing the pharmacologically selective designer Gi-coupled muscarinic receptor (hM4D) along the dorsal-ventral extent of MEC in transgenic mice with GCaMP6s expression under the CaMKIIa promoter (Figure S3; see Methods). Using in vivo two-photon Ca^2+^ imaging, the results show that the frequency of Ca^2+^ transients was reduced by 80% at 30 minutes post 1 mg/kg DCZ injection compared to baseline (n=302 neurons across 2 mice; P < 0.01 – KS test) (Figure S3b). In order to test whether MEC activity is necessary for tDNMS learning, we bilaterally injected AAV expressing hM4D across the dorsal-ventral extent of MEC (Figure 4b). Mice then underwent water restriction and shaping before beginning the tDNMS task. We monitored learning across 8 sessions of the tDNMS task and administered the DREADD agonist DCZ via I.P. injection 5 minutes prior to each session to inhibit MEC for the duration of training. As expected, control mice learned the task well within 8 days, averaging 72.76 ± 2.62% correct responses across sessions 7-8 (Figure 4c) (Repeated Measures ANOVA F_7,15_ = 5.09, p < 0.001). In contrast, DREADD mice showed no improvement from session 1 (54.07 ± 3.79% correct responses) to sessions 7-8 (59.10 ± 3.28% correct responses) (Repeated Measures ANOVA F_7,14_ = 1.18, p =0.32). Inactivation of MEC completely abolished the ability of mice to learn the tDNMS task.

To better understand the specific deficit caused by MEC inhibition, we analyzed data by trial type. We first noticed that control and DREADD mice perform well on short-long trials, starting immediately on session 1 (Figure 4e). This is likely due to learning that took place during the shaping phase of the task (before DCZ injections), as mice were presented with nonmatch trials. Interestingly, we noticed that performance on Day 1, for both control and DREADD mice, was poor on long-short nonmatch trials, even though mice were also presented with these trials during shaping. Drawing upon prior work on timing behavior^31,32^, we suspect the relatively poor performance on long-short trials was due to impulsive behavior driving mice to lick at the offset of the long odor presentation. Notably, we found that learning in the tDNMS task in control mice is largely driven by an improvement in matched trials (Figure 4e; 44.16 ± 4.34 % correct matched trials on Session 1 vs. 69.27 ± 5.01% correct matched trials on Session 8; Repeated Measures ANOVA F_7,15_ = 5.93, p <0.001). We therefore expected DREADD mice to show impaired learning in this trial type. Interestingly, DREADD mice showed an initial improvement in matched trials over sessions 1-3 (40.43 ± 4 % correct matched trials on Session 1 vs. 56.31 ± 4.17% correct matched trials on Session 3; Repeated Measures ANOVA F_2,14_ = 8.01, p <0.01), which is reflected in their overall learning curve. However, matched trial performance leveled near 50% (50.69 ± 3.63% correct matched trials over sessions 6-8), indicating that mice guessed whether to lick or not. While control mice clearly learned to withhold licking in matched trials, DREADD mice perseverated with licking behavior (Figure 4d). Notably, both DREADD and control mice engaged in predictive licking on both types of nonmatched trials from sessions 1-8 (Figure 4d). This indicates that MEC inhibition did not affect ability to perceive or estimate learned durations. The key behavioral difference between control and DREADD mice was that DREADD mice perseverated on rigid behavioral strategies and failed to adopt a flexible strategy to solve the task. MEC inhibition specifically prevented mice from learning to use temporal relationships, or temporal context, to flexibly guide behavior.

An alternative explanation for our results is that MEC inhibition may not affect the learning of temporal context, but rather, other aspects of behavior necessary for tDNMS performance. We therefore performed addition analyses to determine whether MEC inactivation impacted non-timing behavior. Our first concern was that MEC inactivation may impair odor perception. However, the strong performance and predictive licking shown by DREADD mice on nonmatch trials clearly indicate intact odor perception (Figure 4d,e). Our next concern was that MEC inhibition simply made mice more impulsive. Mice must refrain from licking to succeed in the task. If mice are impulsive, they would struggle to withhold even if they understood the task. To address a potential effect of differential impulsivity between control and DREADD mice, we compared the average time from trial onset to first lick for both control and DREADD mice across session 1. If MEC inhibition caused mice to be more impulsive, DREADD mice should lick earlier. This was not the case, indicating MEC inhibition did not make mice more impulsive (Figure S3c). Thus, our data confirm that MEC inhibition causes a specific deficit in learning flexible timing behavior.

### MEC is not necessary for ongoing tDNMS performance

The emergence of context-dependent timing activity led us to test, and confirm, the hypothesis that MEC is necessary to learn flexible timing behavior. To determine whether MEC has an ongoing role in driving flexible timing behavior after learning, following 8 consecutive sessions of MEC inactivation, we took mice off DCZ and instead administered saline over sessions 9-14, then returned to DCZ administration to inhibit MEC over sessions 15-16 (Figure S4a). As expected, without MEC inhibition, DREADD mice learned the task (Repeated Measures ANOVA F_5,8_ = 3.72, p <0.01), reaching 73.65 ± 2.9% correct responses over sessions 13-14 (Figure S4b). Interestingly, there was no difference in behavior from sessions 13-14 to 15-16, showing that MEC is not required to perform the tDNMS task after learning (Figure S4b).

### MEC is not required to learn rigid timing behavior

The emergence of context-dependent MEC time cells and clear necessity of MEC activity in learning the tDNMS task support the hypothesis that MEC has a specific role in flexible timing behavior. However, an alternative possibility is MEC is necessary to learn any new timing behavior, even rigid behaviors. To disentangle these possibilities, we trained DREADD and control mice on a rigid, fixed interval task, in which a drop of water was delivered to the head-fixed mouse every 10s^16^. Mice engage in predictive licking, defined by an increase in licking leading up to reward delivery, when they learn the temporal structure of the task (Figure S4c). To test if MEC is needed to learn this simple timing task, we administered DCZ prior to each of 5 training sessions to inactivate MEC. DREADD and control mice equivalently engaged in predictive licking, indicating clear knowledge of the task structure (Session 5-Control mice predictively licked on 57.76 ±7.27% of trials, DREADD mice on 70.88 ±4.39% of trials, p = 0.15 in unpaired t-test; Repeated Measures ANOVA Group Type x Time F_4,4_ = 1.98, p = 0.11). (Figure S4d,e). Therefore, MEC is not needed to learn rigid temporal relationships, indicating a specificity in flexible timing behavior.

### Pairwise activity of time cells is maintained during non-task epochs

**Figure 5.**
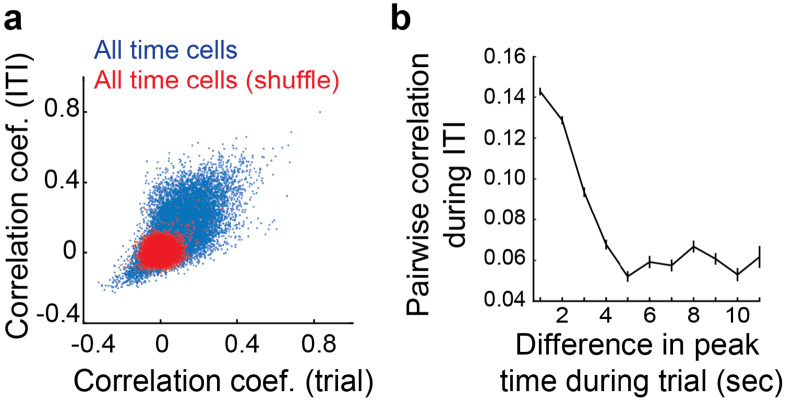
Activity correlation between time cells maintained across task and non-task epochs. **a.** Pairwise correlation between all time cells during the tDNMS task and during the ITI for real (blue) and shuffled data (red). **b.** Pairwise correlation of MEC time cells during the ITI as a function of their relative peak timing field activity during the tDNMS task. (mean ± sem)

There has been a growing body of evidence that a continuous attractor network (CAN), mediated by local recurrent synaptic connectivity in MEC, drives the neural dynamics of spatially selective grid cells^59-62^. Given the strong experimental support for this model in MEC, we wondered whether MEC time cells might also be driven through the same CAN mechanism. In this model structured recurrent synaptic connectivity drives an “activity bump” in a local subpopulation of neurons. This activity bump is then translated across the network as a function of feedforward input which results in regular phasic activity among neurons in the CAN. A strong prediction of this model is that the relative phasic activity of cells in the network should be coherent during task and non-task relevant epochs. To test this prediction of the CAN model, we first measured the pairwise correlations of MEC time cell activity during the tDNMS trial epoch and compared these to the pairwise correlations when mice were not actively timing during the ITI. We find that the coherence of pairwise activity between trial period and inter-trial interval (ITI) is strongly positively correlated and much higher than the chance level (Pearson’s r = 0.63 in actual, r = 0.10 in shuffle, n = 21396, z = 66.7, p < 0.001; Figure 5a). Next, we sorted MEC time cells according to their relative phases during the tDNMS task and computed the pairwise correlation between MEC time cells during the ITI as a function of the time difference in the peaks of their firing fields in the tDNMS task. The results demonstrate that pairs of MEC time cells that are active around the same time in the tDNMS task also display high probability of co-firing during non-task epochs, (Pearson’s r = −0.26, p = 0; Figure 5b). These results are consistent with key predictions of a local recurrent CAN model that may support the regular, sequential activity of MEC time cells during timing behavior.

## Discussion

The ability of the MTL memory system to encode context allows animals to form and recall memories of specific instances, so that memories of individual experiences can be used to flexibly guide behavior. While MTL structures are well-known to encode spatial context, it has remained unclear whether MTL structures are involved in distinguishing temporal context during timing behavior, and if so, how. In this study, we sought to determine whether MEC has a role in flexible, context-dependent timing behavior. We have taken inspiration from pioneering studies that used the delayed nonmatch to sample paradigm to give early insights into a specific role for medial temporal lobe structures in learning and memory^25-27^. By developing and applying a temporal variant of this task, the results from our in vivo neurophysiological recordings and neural manipulation experiments offer insight into a special role of MEC for flexible interval timing behavior. A key finding of this study is that the neural dynamics of MEC time cells become context-dependent over the course of learning. Several observations provide evidence to suggest that the learned dynamics support learning of complex timing behavior. First, at specific moments in the timing task, when the animal can disambiguate temporal context, the population dynamics diverge, with different subpopulations of MEC time cells becoming active for the reminder of the trial. Second, we find that over learning, the population of MEC time cells shifts to over-represent later times in the trial, when the animal can disambiguate trial type, and thereby solve the instrumental task. Third, on error trials, when the animal mistakenly classifies the trial context, we find that the time-locked sequence of MEC time cells is disrupted. Finally, we see that this role for MEC in learning flexible interval timing behavioral may be unique. We find that mice are completely unable to learn this task with MEC inactivation. Importantly, mice are capable of some forms of timing behavior despite MEC inactivation: MEC inhibition does not impair learning of a temporal interval (Figure S4) or timing of previously learned durations, as demonstrated by the presence of predictive licking on nonmatched trials (Figure 4d). These results point to a specific necessity of MEC in flexible, context-dependent timing behavior. Overall, our findings are in line with prior work demonstrating inactivation or lesions to MEC can disrupt timing behavior ^18,44,45^. Notably, the large effect we have observed in the novel tDNMS task (Cohen’s d = 1.13 for the % change in performance from session 1- 7&8 for control and MEC inactivation mice), supports the idea that more flexible and/or complex timing behavior relies heavily on MEC compared to simple tasks where effects are small or nonexistent. That is, MEC is not necessary for learning more rigid timing behavior, which can likely be learned through basal ganglia structures ^10,11,13,15-17^. Together, our results suggest that MEC could play a specific role in learning flexible interval timing through regular sequential activation of context dependent MEC time cells.

This finding of timing activity in MEC stands in contrast to the vast majority of studies which have focused on a specialized role for MEC in navigation and spatial memory^20,24,33-41^. In our task, mice were free to run on a cylindrical treadmill and often exhibited stereotyped running and approach behavior at specific moments when they were able to discriminate nonmatch-go and match-go trials (Figure S2d). To rule out the possibility that we were in fact measuring distance, rather than time, coding cells we made several measures that consistently demonstrate MEC time cells were in fact yoked to elapsed time, but not distance (Figure 2h). Moreover, we find that many MEC time cells fired selectively for specific nonmatch trial types (long-short vs short-long), when the running and approach behavior were identical, further suggesting timing and temporal context were the salient behavioral variables (Figure 2f; Figure S2d). As a third measure, we also examined velocity tuning of MEC time cells. Across the population of MEC time cells there was a small but significant correlation between the DF/F activity and velocity (0.0022 ± 0.0046 median ± SEM; P<0.035 Sign Rank Test) (Figure S2b). However, the distribution of correlation coefficients for MEC time cells was not significantly different from non-time cells (P=0.82; Wilcoxon Rank Sum Test) (Figure S2b), suggesting velocity cannot account for the significant time-locked activity seen in MEC time cells. Together, with the literature on spatial navigation and a recently expanding literature demonstrating a role for MEC in nonspatial memory, data support a broader role for MEC in mnemonic function ^6,29,42,43^.

Interval timing is a fundamental computation that is necessary for many adaptive behaviors and cognitive processes, including learning and memory. Yet, prior work to investigate a role for the entorhinal cortex and the hippocampus in temporal processing has been focused largely on questions of how memories evolve over time or how information is linked across disparate temporal epochs^4,46-49^, but not interval timing per se. Our novel timing paradigm used in this study offers insights not only into neural dynamics, but also the cognitive strategy that may support the learning of flexible timing behavior. In our version of the tDNMS task, mice were presented with three trial types: short-long and long short, which served as nonmatch-go context, and short-short trials, which served as the match-no-go context. Importantly, long-long match trials are absent in this paradigm, and the long duration stimuli are therefore only presented on nonmatch-go trials. Using this three trial type structure permits subjects to employ one of several potential cognitive strategies to discriminate match-no-go and nonmatch-go context on each trial (see Methods for more details on distinct strategies). Together, the data showing that 1) manipulating the interstimulus interval (Figure S1 and Figure S1f) does not change the timing of predictive licking to reward or decrease % correct on probe trials, 2) predictive licking is present, especially on long-short trials (Figure 4d) and 3) the distribution of predictive lick times, relative to reward delivery, does not change on interstimulus interval probe trials (Figure S2d), suggests that mice use the strategy of identifying long stimuli as a go cue, and also time the duration of each consecutive stimulus to predict when to respond (lick) or withhold response on nonmatch or match trials, respectively. We argue that is a relatively complex cognitive process that requires encoding a hierarchical structure between multiple duration stimuli on each trial and learning to respond at specific predicted moments in this task structure.

Since the discovery of spatially selective MEC grid cells there has been a strong focus on the role of MEC in navigation and spatial memory. This research program has been remarkably productive, and although there still are some outstanding questions remaining, this line of research has given substantial insight into the circuit mechanisms that underlie grid cell firing. Namely, there is strong support for a CAN that is mediated through structured local recurrent synaptic connectivity of MEC neurons ^59-62^. A key feature to this model is that the network integrates synaptic input coding for animal heading direction and velocity, thereby driving sequential activity of a population of grid cells, each coding for different spatial phase(s) within an environment. This process is mathematically equivalent to path integration ^59,63^, giving rise to a measure of distance travelled from a start location. We find this computation conspicuously similar to that of a clock, which rather than a measure of distance, can integrate a constant input to give rise to a measure of duration from a start time. When animals are moving through space, their distance and duration travelled are proportional via velocity. Given the environmental context, it could be optimal to estimate different features of elapsed time and/or distance travelled. For example, to minimize the path/duration to home from a patch location, animals might sometimes use the shortest, most direct path while other times it might be optimal to compute the minimal duration across multiple routes. As strong prediction of the CAN model is that neurons encoding similar phases while engaged in a relevant behavioral task, such as firing at similar locations in space during navigation or similar delay times during interval timing, will display coherent phasic activity during non-task relevant epochs such as the ITI. Consistent with this prediction, we find that the correlational structure of MEC time cells, as defined during the tDNMS task, remains coherent during the ITI when the animal is not actively timing. We suspect that during timing, the sequential activity of MEC time cells is driven through similar continuous attractor network dynamics, that may have evolved for similar and often overlapping navigation processes across time and space. Such overlap could indicate that MEC neurons may serve as a general integration circuit, calculating either distance or time based upon relative behavioral demands.

## Materials and Methods

### Surgery and Behavior

#### AAV injections:

To inhibit MEC, C57-BL6 mice (n = 40 mice: 20 male & 20 female; postnatal 2-3 months) were injected bilaterally with pAAV-CaMKIIa-hM4D(Gi)-mCherry (Addgene: AAV8; 2.40 x 10^13^ GC/mL; diluted 1:1 in PBS) or pAAV-CaMKIIa-mCherry (Addgene: AAV1; 1.40E x 10^13^ GC/mL; diluted 1:1 in PBS). A Nanoject III Injector (Drummond) was used to inject 80nL of virus (divided into 4x20 nL injections, injected at a rate of 10 nL/s) at 6 sites in each hemisphere. Injections were targeted at 2.9mm lateral from bregma and 0.15mm rostral of the transverse sinus; at 3.3mm lateral from bregma and 0.15mm rostral of the transverse sinus; and at 3 depths (1.2mm, 1.6mm, and 2mm) from the dorsal surface of the brain.

#### MEC microprism implant:

All experiments were approved and conducted in accordance with the University of Utah Animal Care and Use Committee. Methods for MEC microprism implant have been described previously^6,64^. Briefly, 8 mice (6m and 2f; 6 for in vivo imaging the tDNMS task and 2 for chemogenetic DREADD validation) C57-BL6 mice (~P70) were anesthetized using 1–2% isoflurane. An approximately rectangular craniotomy was made over the dorsal surface of the cortex (above MEC) and cerebellum with corners positioned as follows: (i) ~2.1 mm lateral of bregma, ~4.5 mm caudal of bregma (~300–500 μm rostral of the transverse sinus); (ii) ~4.5 mm lateral of bregma, ~4.5 mm caudal of bregma (~300–500 μm rostral of the transvers sinus); (iii) ~2.1 mm lateral of bregma, ~7.75–8 mm caudal of bregma (~3.25–3.5 mm caudal of the transverse sinus); and (iv) ~4.5 mm lateral of bregma, ~7.75–8 mm caudal of bregma (~3.25–3.5 mm caudal of the transverse sinus). After the skull was removed, a portion of the cerebellum was aspirated to expose the caudal surface of the cortex. The tentorium separating the cerebellum and cortex was carefully removed, leaving the dura of the cortex completely intact. A microprism (right-angle prism with 1.5-mm side length and reflective enhanced aluminum coating on the hypotenuse, Tower Optical) was mounted on a custom stainless-steel mount (using UV-curable adhesive, Norland). This assembly was then positioned by aligning the front face of the microprism parallel to the caudal surface of the MEC and aligning the top surface of the microprism perpendicular to the (eventual) axis of excitation light propagation. A thin layer of Kwik-Sil was applied to the caudal MEC surface before microprism implantation to fill the void between the brain and the front surface of the microprism. The microprism and mount were rigidly held in place and the craniotomy sealed by application of a thin layer of Metabond to all exposed sides of the microprism (except the top surface of the prism) and mount and on any exposed skull or brain. A titanium headplate (9.5 mm × 38 mm) was then attached to the dorsal surface of the skull, centered upon and aligned parallel to the top face of the microprism. A titanium ring (27-mm outer diameter and 12.5-mm inner diameter, with a 3-mm high edge) was then attached to the top surface of the headplate, centered around the microprism, and the area between the craniotomy and the inner edge of the metal ring was covered with opaque dental cement (Metabond, Parkell, made opaque by adding 0.5 g of carbon powder, Sigma Aldrich).

#### Experimental Setup:

Mice were head-fixed over a cylindrical treadmill (60 cm circumference and 10 cm width), which was enclosed in a box (60 cm length x 60 cm width x 63.5 cm height). After being head-fixed, an odor nozzle and lick spout were quickly placed near the mouse. Odorized air was delivered using a flow dilution olfactometer (Verhagen et al. 2007). The olfactometer consisted of two streams of air: a carrier stream (0.9 L/min) and an odorized stream (50 mL/min) which carried isoamyl acetate (2% isoamyl acetate in mineral oil; odorant from Cole-Parmer, 99+%). The two streams combined, and a solenoid valve was used to direct the odorized airflow either to the mouse (via the odor nozzle) or to a vacuum (1.8 L/min). Odor delivery was validated using a photoionization detector (PID). Licking was monitored throughout each training session and was detected using a capacitance sensor (SparkFun Capacitive Touch - AT42QT1010) with an electrode positioned on the lick spout. A solenoid valve was used to deliver water (~6 ul per drop) via the lick spout when appropriate. All experimental paradigms were automated and controlled using an Ardunio Uno, and data collection was performed using a Picoscope oscilloscope (PICO4824, Pico Technology, v6.13.2) sampling at 1kHZ. Mice were free to run on the treadmill during all training sessions, and mouse velocity was measured using a rotary encoder (E2-5000, US Digital). All training was performed in the dark, during the dark phase of the animals’ light cycle.

#### Behavioral Training:

After recovering from surgery, mice began water restriction (~1 mL of water per day). Once mice reached ~85% of their initial weight, they began pre-training for the tDNMS task. Mice were first acclimated to the experimental setup though a habituation phase. During habituation, series of 50 drops of water (3s apart) were delivered to the mouse (3-6 series of 50 drops per session). Habituation ended after mice licked to consume ≥80% of water drops in a series of 50 drops. Following habituation, mice began 3 phases of shaping: Shaping 1, Shaping 2, and Shaping 3. Shaping followed the same trial structure of the tDNMS task; however, only nonmatched trials were used. Each trial consisted of a flash of green light to alert mice the trial was about to begin, the first odor, an interstimulus interval, the second odor, and a response window. Trials were separated by a random intertrial interval (ranging from 16-24s). In Shaping 1, a drop of water was automatically delivered 0.25s after second odor offset in each trial. Once mice licked to consume drops in ≥80% of trials, they progressed to Shaping 2. Probe trials were introduced in Shaping 2. During probe trials, the mouse had to lick within a 3s response window following second odor offset to trigger a reward. If the mouse successfully triggered reward, the next trial was another probe trial. If the mouse failed to trigger a reward in a probe trial, the following trial was automatically rewarded, after which the mouse was given another probe trial. Training on this phase continued until mice licked to earn reward in ≥20 consecutive trials. Mice then began Shaping 3, which had the same probe trial format as Shaping 2. However, in addition to licking in the response window, mice were also required to withhold licking during the first odor and interstimulus interval (ISI) to trigger reward delivery. Training on this phase continued until mice reached 2 consecutive sessions of ≥ 20 consecutive rewarded trials, after which they began the tDNMS task. Some mice failed to reach this benchmark yet routinely performed above chance on probe trials. These mice instead began the tDNMS task after reaching ≥ 80% correct performance on probe trials. Following shaping, mice began the tDNMS task, where matched trials were introduced. Matched and non-matched trials were included in a pseudo-random manner and balanced so that half of trials were matched and half were nonmatched. Nonmatched trials were evenly split between short-long and long-short trials. Mice were rewarded in nonmatched trials only if they withheld licking during the first odor and ISI and if they licked within the 3s response window following second odor offset. Mice received no reward in matched trials and were punished with an increased ITI (+12s) for licking in the response window. Mice were trained for 1 session per day, with each session consisting of 100 trials for shaping, or 90 trials for the tDNMS task. Mice were trained at least 5 days/week during pretraining and 7 days/week during the tDNMS task.

MEC inactivation experiments were performed in two cohorts of mice. Each cohort contained a balanced number of Control and DREADD mice, with the experimenter blind to experimental condition. Following tDNMS training, the first cohort of mice was trained for an additional session of 30 trials with no odor (mineral oil only) to ensure mice use odor to solve the task. The second cohort of mice was instead trained on a Fixed Interval (FI) task^16^ following tDNMS training to determine if MEC is necessary to learn more rigid timing behavior. The same experimental apparatus was used for the FI task with the exception of odor delivery. In each session of the FI task, a (~6ul) drop of water was delivered to the head-fixed mouse every 10s (150 drops per session). Mice were trained for 1 session per day for 5 days on the FI task.

Imaging data was collected from separate cohorts of mice. Imaging was performed using transgenic mice expressing GCaMP6s under the CaMKIIa promoter. Two mice were used to validate the efficacy of DREADD-mediated inhibition in MEC and were not trained on the tDNMS paradigm, while all other mice underwent tDNMS training. To determine whether time cells encode absolute or relative time, mice were tested on probe sessions following tDNMS learning in which the ISI was lengthened from 2s to 5s on a subset of trials.

A final cohort of mice was trained on a version of the tDNMS task with modified durations. Due to the three trial-type design, mice could solve the tDNMS task by learning to lick if total trial duration is long and to withhold if total trial duration is short. Standard experiments were performed with a 2s short odor, 3s ISI, and 5s long odor, making nonmatched trials 10s and matched 7s. To test if mice used a rigid strategy, we decided to introduce probe trials that cause matched and nonmatched trials to be the same overall duration. With our standard durations, this would mean lengthening the ISI on matched probe trails by 3s. However, this change would increase task difficulty (increasing the time the mouse has to resist impulsivity & increasing working memory demand), making it difficult to determine if a potential drop in performance is caused by increased task difficulty or use of a rigid strategy. To avoid this problem, we instead trained a separate cohort of mice on a version of the task with 3s short odors, 5s ISI, and 6s long odors so that we could reduce the ISI in nonmatch probe trials to equal total trial duration of matched trials.

#### Two-photon imaging of MEC neurons:

After mice were pretrained on the tDNMS task (14.5 ± 3.9 days for pretraining), we began two-photon laser resonance scanning of populations of neurons expressing GCaMP6s through the microprism using a Neurolabware microscope. Data was acquired with an 8 kHz resonant scanner, images were collected at a frame rate of 30 Hz with bidirectional scanning through and Scanbox software was used for microscope control and acquisition. A Ti:Sapphire laser (Discovery with TPC, Coherent) light at 920 nm was used as the excitation source with average power measured at the sample (after the objective; 20×/0.45 NA air immersion objective (LUCPanFL, Olympus) with correction collar set at 1.25 ) was 50-120 mW. Imaging data was recorded using a PicoScope Oscilloscope (PICO4824, Pico Technology, v6.13.2) sampled at 1kHZ to synchronize with behavior.

### Data analysis

#### General:

Imaging data was analyzed on a IBuyPower Intel Core with Windows10 and custom software written in Matlab (2018b). No statistical methods were used to predetermine sample sizes. Sample sizes were based on reliably measuring experimental parameters while remaining in compliance with ethical guidelines to minimize the number of animals used. Repeated Measures ANOVA, paired t-test, Wilcoxon rank sum test, two-sample Kolmogorov-Smirnov test, chi-squared test, Wilcoxon signed-rank tests were used to test for statistical significance when appropriate, and all statistical tests were two-sided unless stated otherwise. For tests assuming normality, data distributions were assumed to be normal, but this was not formally tested. Experimenters were blinded during data collection of MEC - DREADD inactivation experiments in the tDNMS task (experimenters were not blinded for fixed interval experiments). All data in the text and figures are labeled as mean ± s.e.m. unless stated as mean ± s.d.

#### Behavioral Performance:

Performance on the tDNMS task was analyzed by determining the percent of trials in which mice behaved correctly. Correct nonmatched trials were defined as those in which mice withheld licking during the first odor and ISI and licked in the 3s response window following second odor offset to trigger reward. Correct matched trials were defined as those in which mice withheld licking for the duration of the trial. Mice that met the criteria to advance beyond shaping and begin the tDNMS task were included in analysis. However, a subset of mice were removed from analysis due to: a failure to retain the task structure learned in shaping (performance >3 SD below the mean on tDNMS session 1, n =1 mouse), headplate falling off (n = 1 mouse), or lack of virus expression (n = 2 mice). Though most behavior is analyzed as % correct trials, licking behavior was further examined in some instances. Licking was analyzed by identifying lick events, defined as samples (sampling rate of 1 kHz) in which the capacitance sensor detected a signal. For tDNMS behavior, any licking following reward delivery was removed to focus on predictive and not consummatory licking. Lick events were then binned in 0.25s bins, then normalized to the maximum within the session. Lick events were also used to examine performance in the Fixed Interval (FI) task. Performance on the FI task was measured as the % of trials in which mice engaged in predictive licking, where predictive licking is defined as an increase in lick events (determined by a significantly positive slope) in the 5s preceding reward delivery.

Strategies for solving the tDNMS task: different cognitive strategies used in the tDNMS may differ in their complexity and ability to flexibly generalize. Notably, each strategy makes specific predictions regarding the timing behavior in the standard task and different predictions for how mice should respond to particular types of trial structure manipulations (i.e. probes). Strategy 1: In the most rigid and simple strategy, mice could learn to selectively identify long duration stimuli as a go cue, and wait to respond until the 2^nd^ stimuli offset. This strategy only requires timing for a single interval and predicts that mice should not predictively lick near trial offset times. Strategy 2: Since the nonmatch trials are longer in total duration than the match trials, mice employ a different cognitive strategy to time the full trial interval, and ignore the relative intervals in the first and second stimuli. Again, this strategy only requires timing a single interval. This strategy predicts that mice should withhold licking on a small random subset of probe trials where the interstimulus interval is changed such that the total duration on nonmatch is the same as prior for the match trials. Strategy 3: Mice may also use the strategy of identifying long stimuli as a go cue, and also time the duration of each consecutive stimuli to predict when to respond (lick) or withhold response on nonmatch or match trials, respectively. In this case, mice actively time multiple stimuli durations on each trial, but may not necessarily be comparing their durations to decide to respond or not. A key prediction of this strategy is that mice display predictive licking on nonmatch trials, especially on the long-short trials where the go cue has already been discriminated early in the trial. Further, if the mice are timing the second stimuli on long-short trials, then manipulations of the interstimulus interval on these trials should not change the distribution of predictive licking aligned to the start of the second stimulus. Strategy 4: mice may also learn a general rule to encode both stimuli durations, and compare at the end of the trial to employ the go-nonmatch/No-go-match rule, which is perhaps the most abstract strategy between these four. In this case, mice should not predictively lick on long-short trials, as they should not be classifying these trials as nonmatch until after the stimuli have ended. Data presented in this study argue mice are likely using strategy 3 to solve the DNMS task This strategy is more complex than strategy 1 or 2, which could be solved with a simpler decision structure, but likely not as abstract as a true nonmatch to sample comparison as required for strategy 4. In future work, it is possible that by designing tasks that require even more flexible and/or abstract timing strategies that MEC would be required for ongoing performance.

#### Image processing, ROI selection, and transient analysis:

In vivo two-photon data sets were acquired during the tDNMS task (60,000 frames per session). Movies were first motion corrected using whole-frame cross-correlation, as described previously (Heys et al 2014), and the motion-corrected time-series was used for all subsequent analysis. Regions of interest (ROIs) were defined using Suite2P v0.10.1 ^65^. Significant Ca2^+^ DF/F transients were identified using methods described in ^6,64,66^.

#### Defining time cells in the tDNMS task:

Cells with significant tuning at a specific time point during trials were defined based on mutual information^67,68^. Before computing mutual information, Ca^2+^ signals were normalized in each trial by dividing their peak dF/F value to prevent that one or two large transients determine a cell’s activity pattern. Then, dF/F values were averaged across correct trials, and mutual information of averaged dF/F was obtained using following equation for each trial type (e.g., short-short, short-long, long-short):

$$Mutual\;information=\sum_{i}p_{i}\lambda_{i}\;\log_{2}\frac{\lambda_{i}}{\lambda }$$

where i denotes bin, $p_{i}$ is occupancy rate in *i*th bin, $\lambda_{i}$ is dF/F at *i*th bin, and λ is mean dF/F. To test whether the mutual information is sufficiently higher than that from random activity, dF/F in each trial was circularly shifted in random amount, and mutual information was computed with shuffled data. Shuffling was repeated 1,000 times. P-value was defined as a proportion of shuffled mutual information greater or equal to a real mutual information. Cells were classified as time cells if *i*) mean dF/F > 0.03 and *ii*) p-value of mutual information. < 0.01.

#### Comparing correlation between match and nonmatch trial types across day 1 to day N

Time cells in either short-short or short-long trial types were selected, and the Pearson correlation coefficient between these two trial types was calculated in each cell. The same procedure was performed using time cells in either short-short and long-short trial types. All correlation coefficients were pooled and compared between Day 1 and Day N using Wilcoxon rank sum test.

#### Comparing correlation between match and nonmatch trial types across time phase in trials

Time cells in either short-short or short-long trial types were selected. Population vectors were constructed from dF/F of all selected cells at each time bin. Then, the Pearson correlation coefficient between the population vectors of short-short trial type and their corresponding bin’s population vectors of short-long trial type were computed. The same procedure was repeated for the time cells in either short-short or long-short trial types.

#### Decoding trial types using linear discriminant analysis (LDA)

A classifier for each Day N session was built by linear discriminant analysis (LDA) using MATLAB function fitcdiscr. Only correct trials were used in this analysis. Mean dF/F and peak dF/F time of time cells across trials in the training set was an input matrix for fitcdiscr function. Response input (Y input) for this function was either trial types (i.e., short-short, short-long, long-short) or match vs nonmatch of training set trials. The classifier computed from fitcdiscr then fed to predict function to get decoded responses (e.g., short-short type) corresponding to the testing data set. For Figure 3g, the decoding result of short-short trials was plotted in the first block, and decoding result of short-long and long-short trials were plotted in the second and third block, respectively. We applied leave-one-out cross-validation method, so the classification process was repeated as the number of entire trials. In each iteration, a single trial was selected for testing data set and the rest of trials were assigned for training data set. For calculating chance level of decoding accuracy in Figure 3h, when making classifier the response input (i.e., trial types or match vs. nonmatch) associated to training trials was shuffled. The whole process was repeated 1,000 times to make a distribution of decoding accuracy for shuffle data. The boot-strap p-value was defined by a proportion of decoding accuracy in shuffle distribution equal or larger than that of actual data set.

#### Measuring activity coherence in error trials

Due to the low number of error trials in short-long and long-short trial types, this analysis was only applied to short-short trial type. For each cell’s activity, correct trials were divided into two groups using a random subset of trials (Correct A and Correct B). The number of trials assigned to Correct B was set to match the number of error trials. Cell activity was sorted according to the sequence in correct A group and correlations for each cell’s activity were computed for correct A versus correct B (blue) and correct A versus error (red). Trial random sampling was repeated 1,000 times to get a distribution of correlation coefficients. Then, the mean values of these correlation coefficients were taken as the cell’s correlation coefficient values.

#### Comparing variance in time and distance dimension

The dF/F of time cells were redrawn as a function of elapsed running distance from the trial initiation light being turned on. In each trial, the peak location of dF/F was detected in distance dimension. The variance of the peak locations was measured by coefficient of variation. Coefficient of variation is a ratio of the standard deviation to the mean, which gives a standardized measure of dispersion and is used to compare variations between different scales or dimensions. The same procedure was repeated as a function of elapsed time from the light being turned on. Then, coefficient of variation of time cells were compared between distance and time dimension by Student’s Paired T-test.

#### Comparing pairwise activity correlation between trial period and ITI

To measure the stability of network state, pairwise activity of all time cell pairs was compared between trial period and inter-trial interval (ITI)^69^. The entire time series of dF/F in a session (e.g., 120,000 frames) was segmented into 500 ms time bins, and dF/F values were summed within each time bin. Time bins from 1 second before the first odor onset to the second odor offset (11 seconds) were included in trial period, and time bins from 5 seconds after the second odor offset to 4 seconds before the next first odor onset were included in ITI. There is 3~5 seconds gaps between every trial period and ITI so the possibility that the activity from one epoch affects the other was very small. The time bins within these gaps were excluded from the analysis. Kendall’s correlation (τ values) between series of summed dF/F of either trial period or ITI was computed between all pairs of simultaneously recorded time cells. Then, the similarity of network state across trial period and ITI was measured by computing Pearson’s correlation coefficient between sets of corresponding τ values of trial period and ITI (Figure 5a). The dF/F was circularly shifted in a random amount to compute τ values for shuffle data. For Figure 5b, τ values of ITI were plotted as a function of difference in peak time of cell pair during trial period.

#### Histology

Following behavioral experiments, mice were euthanized and the brain was removed and fixed in 4% PFA in 0.1M PBS for ~24 hours. The brains were then rinsed 3x with 0.1M PBS, then stored in PBS for 1+ days before the tissue was sectioned in 50-100 micron sagittal or coronal slices using a vibrating microtome. Free floating slices were then incubated in 0.1M PBS with 0.1% Triton-X for 15 minutes, washed with 0.1M PBS and incubated for 3 hours in a 25:1 solution of 0.1M PBS with 435/455 blue or 530/615 red fluorescent Nissl stain (Invitrogen). Brain sections were imaged and stitched using a VS200 Virtual Slide fluorescence microscope (Olympus) with a 10x OFN26.5, NA 0.40 objective (Olympus).

## Supplementary Material

1
