# Supplementary material for: Medial entorhinal cortex plays a specialized role in learning of flexible, context-dependent interval timing behavior
Source: Res Sq. 2023 Apr 17:rs.3.rs-2681599. Preprint. [Version 1] doi: 10.21203/rs.3.rs-2681599/v1 (PMC10153298; doi:10.21203/rs.3.rs-2681599/v1)
Supplement: 1 [file NIHPPrs2681599v1-supplement-1.pdf]

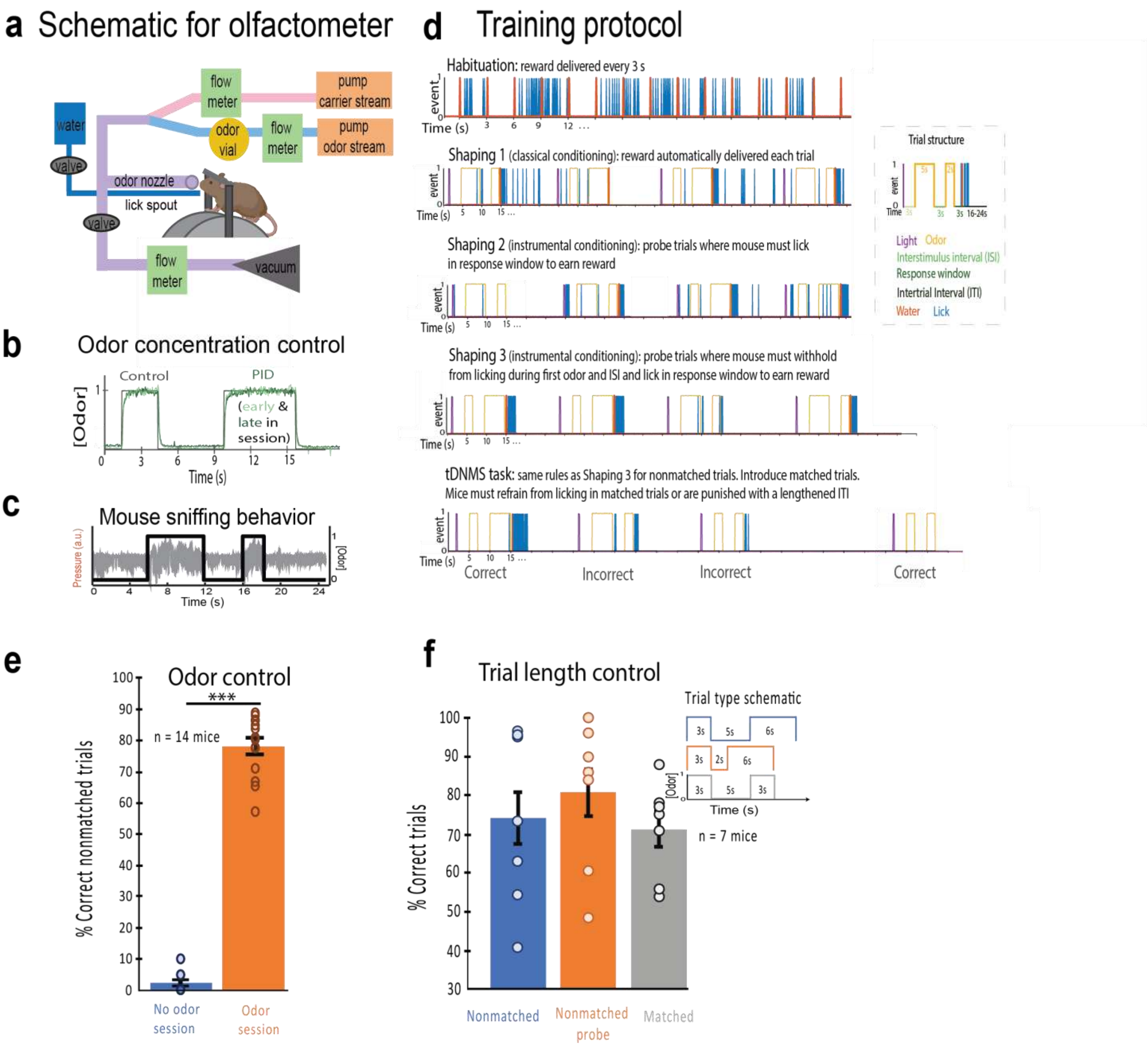

Figure S1. tDNMS Set-Up and Control. a. Experimental set-up. Odorized air is directed either to the mouse or to a vacuum. A lick spout, connected to a capacitance sensor, delivers water and is used to monitor mouse licking. **b.** Odor concentration control. Odor concentration was measured using a photoionization detector (PID). Odor can be delivered with high temporal specificity at a constant concentration over 45 minutes, as shown by PID measurements (green) relative to control signal (black). **c.** Sniffing behavior measured by a flow meter during a sample trial. **d.** Training protocol. Mice undergo three phases of pretraining (see Methods). **e.** Odor control session. After completing tDNMS training, mice were tested with no odorant (mineral oil only). Mice failed to solve nonmatch trials in the absence of odor but performed well in a prior session in which odor was used (2.25 +/- 1.01 % correct responses for no odor condition compared to 78.08 +/- 2.58% correct with odor, $p<0.001$ Student's Paired T-Test; n=14 mice). Bars represent mean +/- SEM. **f.** Trial length control session. The ISI was manipulated on a random subset of nonmatched trials ("probe trials") such that overall trial duration was identical to the matched trial duration. If mice use total trial duration to solve the task, rather than comparing individual stimuli durations, they should incorrectly withhold licking on probe trials. Instead, there was no significant difference between nonmatch (74.14 +/- 6.71%) and probe trial performance (80.71 +/- 6.02%) ($p=0.19$ Student's paired T-test; n=7 mice). Bars indicate mean +/- SEM.

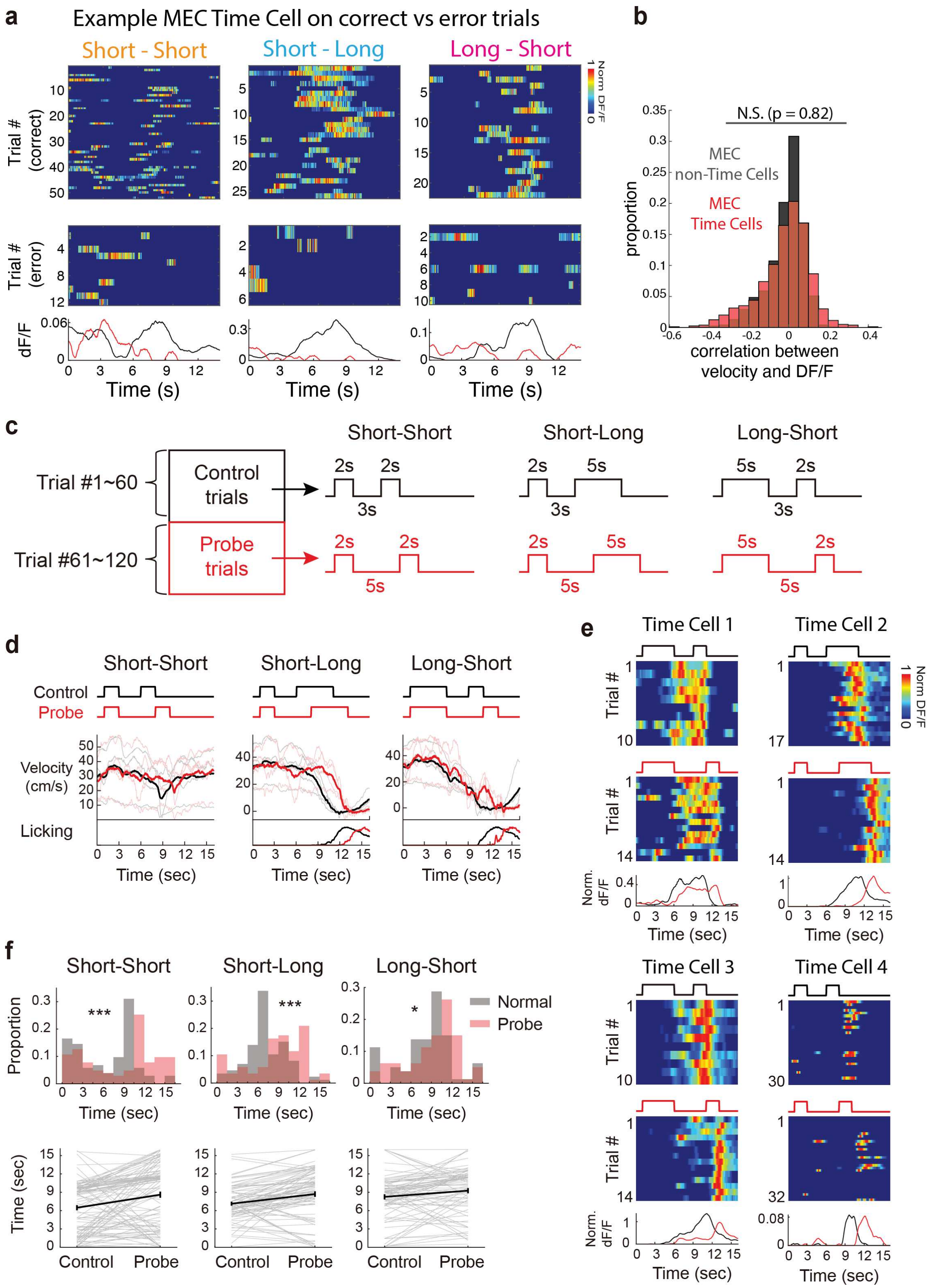

Figure S2. MEC time cells on ISI probe trials. a. Example MEC time cell activity on short-short, short-long and long-short correct (top) and error (bottom) trials. **b.** Histogram of Pearson's correlation coefficients for DF/F versus velocity for MEC time cells (red) and non-time cells (black). **c.** Schematic for probe trials. **d.** Mean velocity and licking on short-short, short-long and long-short conditions during control (black) and probe trials (red). **e.** Four example MEC time cells during control (black) and probe (red) trials. **f.** Time of peak response across the population of MEC time cells on short-short, short-long and long-short conditions during control (black) and probe trials (red).

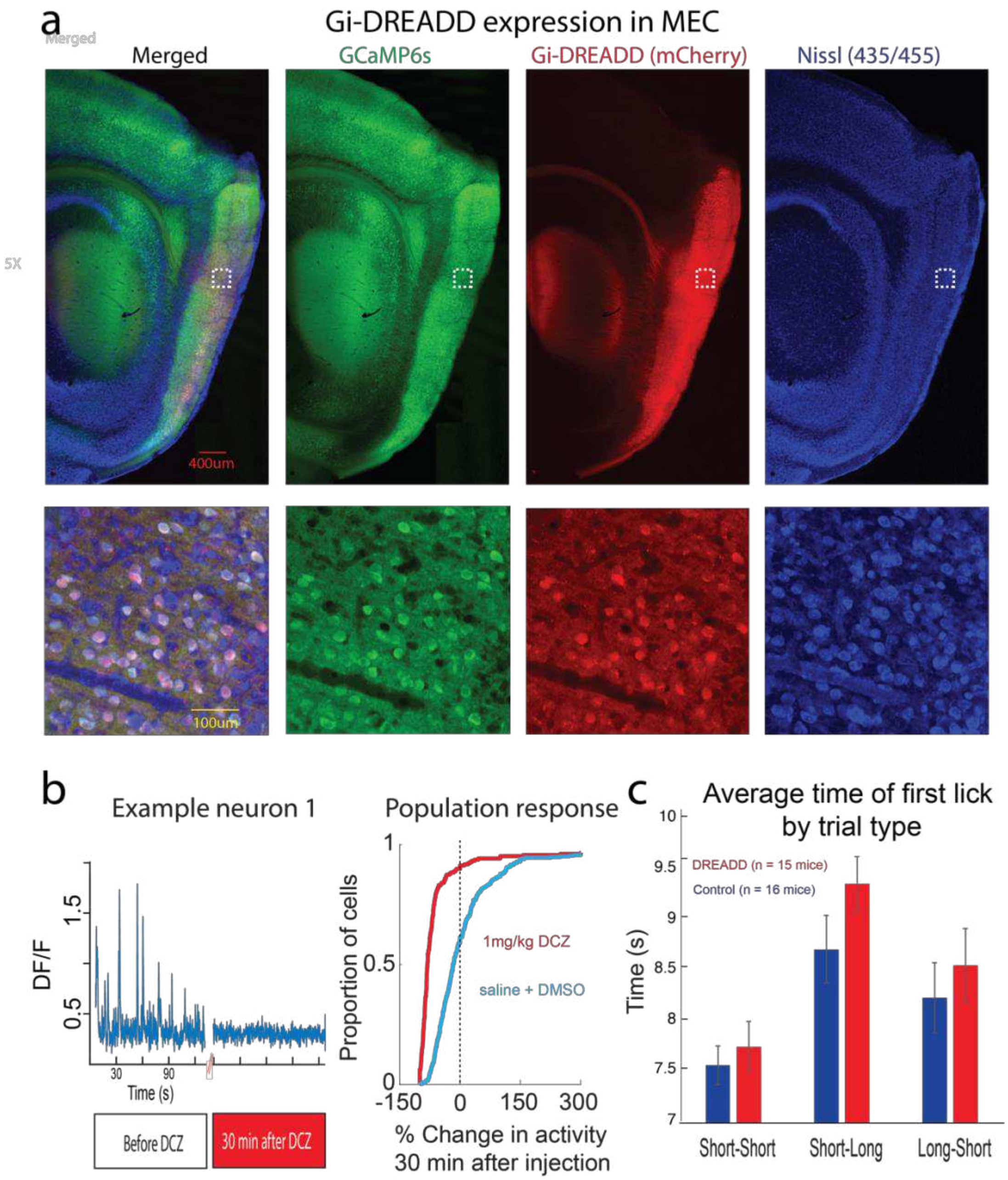

Figure S3. MEC DREADD inactivation. a. Histology showing co-expression of GCaMP6s and hM4D(Gi)-mCherry in MEC. **b.** Simultaneous in-vivo 2-photon GCaMP6s imaging and hM4Di inactivation in MEC. Activation of inhibitory DREADDs by 1 mg/kg IP injection of DCZ reduces average number of Ca2+ transients in MEC neurons by 80% at 30 minutes post injection compared to before DCZ injection. Left, example neuron before and after administration of DCZ. Right, population response. In both the control (blue) and DCZ (red) conditions, GCaMP activity was monitored over 5 minute periods and the change in activity was measured for each cell ($n=302$ neurons in 2 mice; $P<0.01$ Kolmogorov-Smirnov test). **c.** Average time of first lick relative to first odor onset. There is no difference in average time of first lick for DREADD ($n = 15$) and Control ($n = 16$) mice in any trial type (Short-Short: $p=0.56$ Student's unpaired T-test; Short-Long: $p=0.14$ Student's unpaired T-test; Long-Short: $p=0.52$ Student's unpaired T-test).

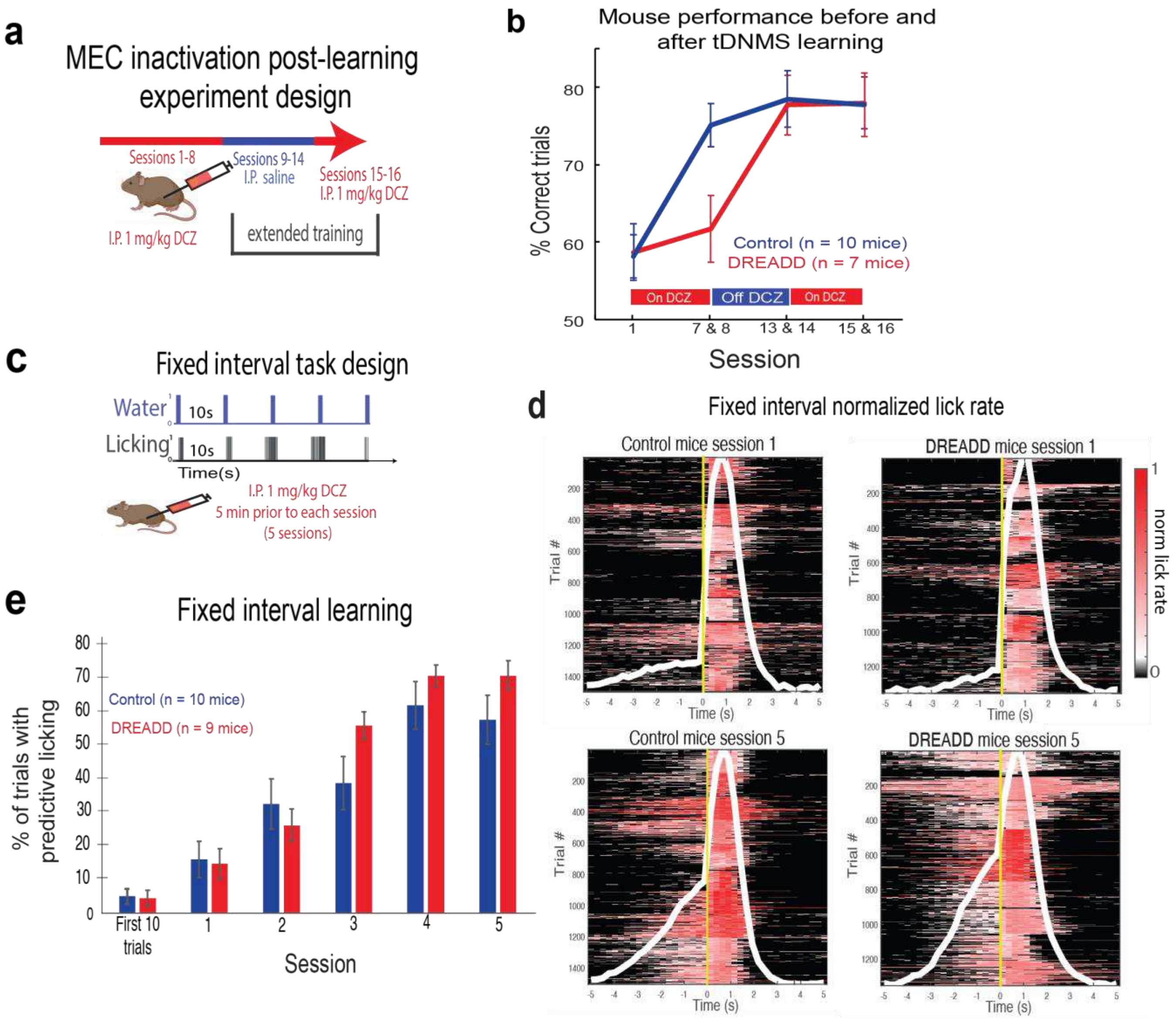

Figure S4. MEC is not required for all interval timing behavior. a. Schematic for inhibiting MEC after learning in the tDNMS task. Following experiments testing the role of MEC during learning (Figure 4), a subset of mice underwent extended training to determine whether MEC activity is necessary for ongoing task performance. **b.** Though MEC inhibition impaired learning in the tDNMS task (Sessions 1-8), DREADD mice learned the task in the absence of MEC inhibition (Sessions 9-14). Following learning in sessions 9-14, subsequent administration of DCZ to inactivate MEC did not affect performance in Sessions 15 and 16 (-0.38 +/- 4.01% change in correct response from sessions 13&14 to sessions 15&16 in Control mice (n=10) versus 2.19 +/- 7.30% change in correct response from sessions 13&14 to sessions 15&16 in DREADD mice (n=7); p=0.74 Student's unpaired T-test). **c.** Fixed interval task schematic. MEC DREADD and Control mice were trained on a fixed interval task (Toda et al. 2017). A droplet of water (4-6ul) was delivered every 10s to head-fixed mice. Licking was measured; time-locked predictive licking indicated learning of timing of water delivery. **d.** Licking behavior of DREADD (n=9) and Control (n=10) mice on sessions 1 and 5 of the fixed interval task. Licking was normalized to the maximum lick frequency with each session for each mouse. All trials for all mice are shown; water delivery occurs at 0s, indicated by line. Average lick response for each session is shown (white). **e.** Fixed interval learning. Predictive licking is defined as an increase in lick rate, measured over 5 seconds preceding the upcoming reward delivery. Percent of trials with predictive licking is not significantly different for Control compared to DREADD mice (Repeated Measures ANOVA Group x Time $F_{4,4}$ = 1.98, p=0.11). Data represent mean +/- SEM.
